# Simulations of Amyloid-Forming Peptides in the Crystal State

**DOI:** 10.1007/s10930-023-10119-3

**Published:** 2023-05-05

**Authors:** A. Najla Hosseini, David van der Spoel

**Affiliations:** Department of Cell and Molecular Biology, Uppsala University, Box 596, SE 75124 Uppsala, Sweden

**Keywords:** Crystal structure, Amyloid fibril, Molecular dynamic simulation, Standard force fields

## Abstract

There still is little treatment available for amyloid diseases, despite their significant impact on individuals and the social and economic implications for society. One reason for this is that the physical nature of amyloid formation is not understood sufficiently well. Therefore, fundamental research at the molecular level remains necessary to support the development of therapeutics. A few structures of short peptides from amyloid-forming proteins have been determined. These can in principle be used as scaffolds for designing aggregation inhibitors. Attempts to this end have often used the tools of computational chemistry, in particular molecular simulation. However, few simulation studies of these peptides in the crystal state have been presented so far. Hence, to validate the capability of common force fields (AMBER19SB, CHARMM36m, and OPLS-AA/M) to yield insight into the dynamics and structural stability of amyloid peptide aggregates, we have performed molecular dynamics simulations of twelve different peptide crystals at two different temperatures. From the simulations, we evaluate the hydrogen bonding patterns, the isotropic B-factors, the change in energy, the Ramachandran plots, and the unit cell parameters and compare the results with the crystal structures. Most crystals are stable in the simulations but for all force fields there is at least one that deviates from the experimental crystal, suggesting more work is needed on these models.

**Supplementary Information:**

The online version contains supplementary material available at 10.1007/s10930-023-10119-3.

## Introduction

The aggregation and deposition of amyloid fibrils, such as amyloid transthyretin and amyloid light-chain, may lead to amyloidosis, which can occur in different organs  [1–4]. Amyloid fibrils such as yeast prion protein Sup35, insulin, Alzheimer’s amyloid-$\beta$, $\tau$, and amylin, contain pairs of tightly bound $\beta$-sheets or “steric zipper” structures, as has been revealed by X-ray diffraction studies [5, 6], for recent reviews on this topic see [7, 8]. Steric zippers are parallel to the fibril axis and account for amyloid aggregations [9]. The stability of these peptides structures is determined by the hydrogen bonds that form along the fibril axis, van der Waals interactions, electrostatic interactions, the hydrophobic effect, and $\pi$-$\pi$ stacking between side chains [10–12]. Experimental as well as theoretical structural studies of amyloids and amyloid-like fibrils have been used to uncover the pathological architecture of amyloid proteins at the molecular level [5, 6, 9, 13, 14]. It is fair to say, therefore, that high-resolution crystal structures of amyloid-forming peptide fragments could serve as a template for designing effective inhibitors [15].

For instance, Seidler et al. have shown that the aggregation-prone segment of $\tau$ with the sequence SVQIVY (present in the cores of patient-derived fibrils) forms steric zipper interfaces. It has been shown that structure-based peptide inhibitors such as VQIINK and VQIVYK can reduce aggregation and toxicity of amyloid-$\beta$ fibrils [16, 17]. Another in-vitro study has suggested the potential of KLVFFA to be used as a template for inhibiting the interaction between amyloid-$\beta$ and a neuronal cell receptor (LilrB2) [18]. On the other hand, it was suggested based on molecular dynamics simulations that the conformation of amyloid-forming peptides in the crystal may not be representative of what is found in solution [19] and it has been hypothesized that the N and Q amino acids may be the reason for this structural polymorphism seen in some amyloid peptides [20]. More in general, there is a competition between fibril formation and crystallization that depends on environmental conditions such as the pH [21]. For a review on the biology of amyloids including structure-based design of therapeutics, please see Chen et al. [22].

Simulation approaches have been used to shed light on the interactions between these peptides using all from simple lattice models to all-atom models with explicit water [23, 24]. From a simulation perspective, crystals are the perfect periodic structure that can be treated with periodic boundary conditions (PBC) without this being an approximation. Accurate PBC simulations should explicitly include long-range electrostatic interactions [25, 26] as well as dispersion interactions [26, 27]. It has been demonstrated in many studies that explicit long range interactions are crucial for accurate simulations [28–30]. In combination with efficient and flexible simulation codes [31], molecular simulations can be used to extract relevant physicochemical properties [32, 33] that can be compared to experimental data [34–37]. In this manner, classical force fields underlying the simulations can be benchmarked against the usually very accurate macroscopic properties that are available in handbooks [38–40]. Although many such benchmarks have been performed for small molecules in the gas and liquid phase, less effort has gone into studying the solid state [41]. In a recent paper evaluating simulations of 30 organic crystals, Schmidt and co-workers found significant deviations from experimentally determined solid densities and sublimation enthalpies [42], suggesting that further scrutiny of force fields for application in the crystal state is warranted. Indeed, Janowski et al. did simulations of the decapeptide “fav8” in the crystal and found that the agreement between simulated and experimental diffraction pattern was not very good [43]. It seems fair to question, therefore, whether simulation methods that cannot reproduce the properties of a molecular crystal can yield accurate results in other phases.

**Fig. 1:**
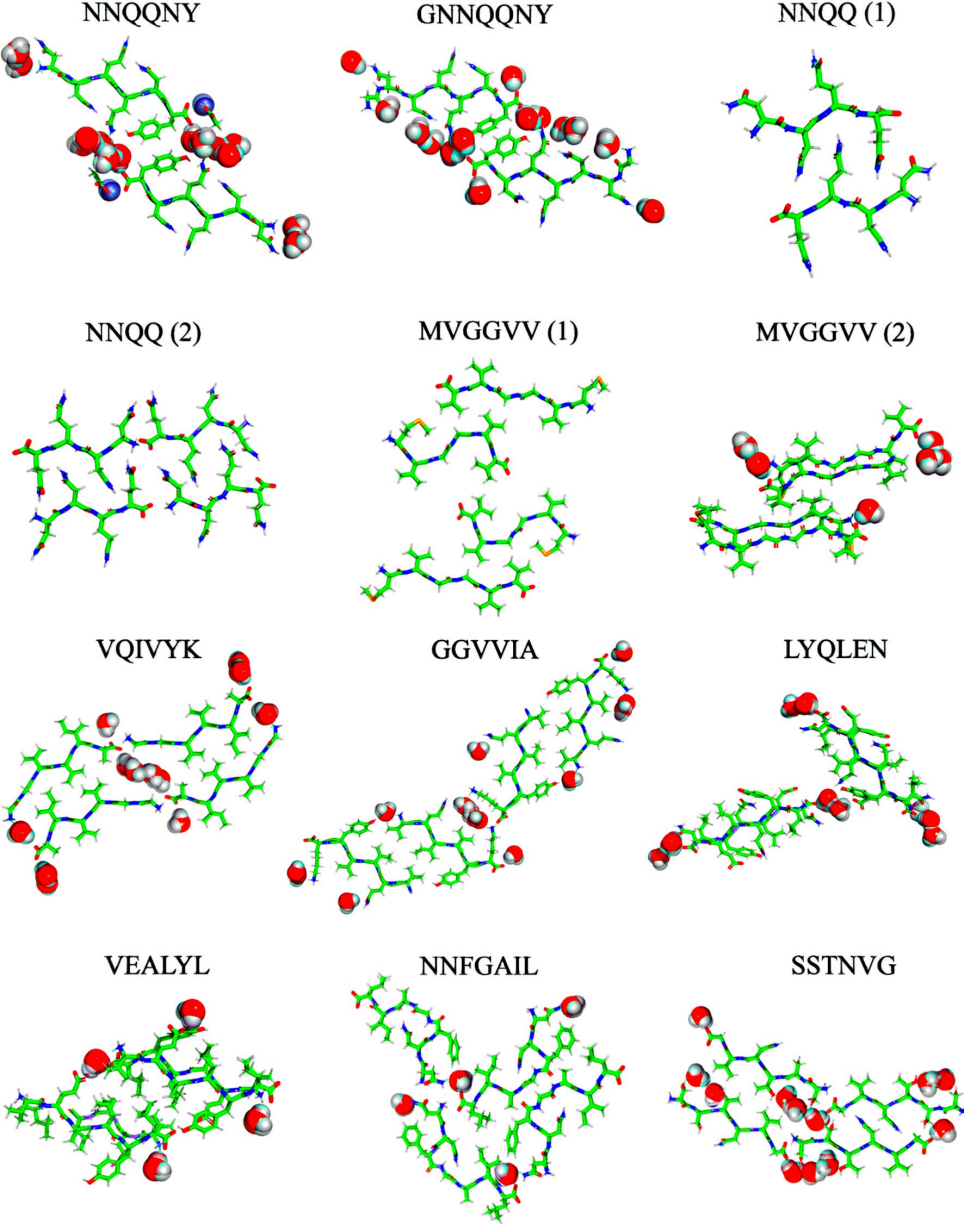
Optimized crystal structures (unit cells) of twelve amyloid peptides with crystallization water molecules. Carbons are colored green, hydrogens are colored white, nitrogens are colored blue and oxygens are colored red. Zinc atoms associated with the NNQQNY peptide are colored purple, and both Zn and water molecules are presented using Van der Waals spheres (Color figure online)

In this study, we present simulations of twelve peptide crystals corresponding to ten different amyloid-forming peptides (see Table 1 and Fig. 1). Simulations are presented at the temperatures used for the diffraction experiments as well as the temperature used for crystallization. We consider three popular force fields, AMBER19SB [44], CHARMM36m [45], and OPLS-AA/M [46]. The peptide crystals contain more or less well-defined water molecules that are important for peptide stability (Table 2). These water molecules are included explicitly in the simulations. Based on the simulation trajectories, we study the evolution of the lattice parameters, the hydrogen bonding between the amino acids as well as between amino acids and water. In addition, we study the dynamics of peptides in the supercells, consisting of 16–64 unit cells, by comparing crystallographic B-factors to the calculated B-factors based on the mean square positional fluctuations (MSF). Finally, we analyze the secondary structure through Ramachandran plots [47].

**Table 1:** Sequence, PDB code, and source of the peptides

Sequence	PDB	Source	Residues	T_exp_	#W	#P
NNQQNY	1YJO [5]	Yeast prion Sup35	7–13	293	14	2
GNNQQNY	1YJP [5]	id.	id.	293	14	2
NNQQ (1)	2ONX [9]	id.	8–11	298	0	2
NNQQ (2)	2OLX [9]	id.	id.	298	0	4
MVGGVV (1)	2OKZ [9]	Alzheimer’s A-*β*	35–40	298	0	4
MVGGVV (2)	2ONA [9]	id.	id.	298	6	4
VQIVYK	2ON9 [9]	Repeat region of *τ* protein	306–311	291	14	4
GGVVIA	2ONV [9]	Alzheimer’s A-*β*	37–42	291	12	4
LYQLEN	2OMP [9]	Human insulin chain A	13–18	310	12	4
VEALYL	2OMQ [9]	Human insulin chain B	12–17	310	4	4
NNFGAIL	3DGJ [48]	Islet Amyloid Polypeptide	21–27	293	4	4
SSTNVG	3FTR [48]	id.	28–33	298	12	4

. Temperature T_exp_ of crystallization, number of crystal waters (#W) and number of peptides (#P) in the unit cell

**Table 2:** Unit cell dimensions and angles for the peptides in this study

Sequence	Unit cell length (Å)			Unit cell angle (°)			M.F.	#P	#W
	a	b	c	*α*	*β*	*γ*			
NNQQNY	21.153	4.87	23.13	90	102.93	90	$2\times 8\times 2$	64	448
GNNQQNY	21.937	4.866	23.477	90	107.08	90	$2\times 8\times 2$	64	448
NNQQ (1)	4.854	16.014	15.546	90	96.91	90	$8\times 2\times 4$	128	0
NNQQ (2)	15.479	4.915	30.552	90	90	90	$4\times 8\times 2$	256	0
MVGGVV (1)	15.148	9.576	23.732	90	96.9	90	$4\times 4\times 2$	128	0
MVGGVV (2)	25.862	9.699	15.851	77.18	74.69	86.93	$2\times 4\times 4$	128	192
VQIVYK	4.863	61.926	15.413	90	98.11	90	$8\times 1\times 2$	128	448
GGVVIA	16.76	41.134	4.789	90	90	90	$2\times 1\times 8$	64	192
LYQLEN	9.666	28.003	17.346	90	96.24	90	$4\times 2\times 4$	128	384
VEALYL	18.425	9.613	21.975	90.88	96.12	100.93	$2\times 4\times 2$	64	64
NNFGAIL	26.19	4.897	31.38	90	90	90	$2\times 8\times 2$	128	128
SSTNVG	16.59	4.789	40.229	90	90	90	$2\times 8\times 1$	64	192

Multiplication factors (M.F.) indicate how many unit cells were stacked to build a simulation box. Total number of peptides (#P) and waters (#W)

## Methodology

### Preparation of the Simulation Supercells

Initial structures were taken from the protein data bank (Table 1). Molecular topologies were created using the parameters from the AMBER19SB [44], CHARMM36m [45], and OPLS-AA/M [46] force fields. Crystal water molecules were parameterized according to the OPC [44] for AMBER19SB and TIP3P [49] for CHARMM36m and OPLS-AA/M, as these are recommended to be used in conjunction with the respective force fields. To create supercells, the unit cells (Fig 1) were replicated using the GROMACS [50] genconf tool, resulting in 16–64 copies of the unit cell (Table 2). The NNQQNY peptide contains a N-terminal acetyl group which was parameterizes using the CHARMM-GUI for AMBER19SB and CHARMM36m [51–53], and the PolyParGen server  [54] for the OPLS-AA/M force field, respectively. A neutral N-terminus was used for the positively charged peptide VQIVYK [55]. A neutral C-terminus was used for peptides with negatively charged side chaina, LYQLEN and VEALYL. For other peptides we have used a charged N- and C-terminus. Coordinate files of the supercells before and after simulation are available from the MDBenchMark github repository [56].

### Computational Methods

All-atom MD simulations were conducted using the GROMACS 2021 package [57]. The systems were equilibrated in the constant volume (NVT) ensemble for 10 ns with position restraints on the heavy atoms with a force constant of 1000 kJ × mol^−1^ × nm^−2^. Separate simulations were performed at the temperatures which was used to grow the crystal fibrils (Table 1) and at 100 K, roughly corresponding to the temperature used for diffraction experiments. The V-rescale thermostat [58] was used with a coupling time constant of 0.1 ps. After minimization with the steepest decent algorithm, the systems were simulated at constant pressure for 50 ns using a 1 fs integration time step. The pressure was maintained at 1 bar using the Berendsen barostat [59] because it is not prone to large fluctuations [50]. A 10 ps pressure coupling time was used for the equilibration and 100 ps for the production runs. Anisotropic pressure coupling in the x-y-z directions with a compressibility of 10^−5^ in all dimensions, including the off-diagonal elements of the pressure tensor, was used for all simulations allowing for the box edges as well as lattice angles to adapt. Chemical bonds to hydrogen atoms were constrained using the linear constraint solver (LINCS) algorithm [60, 61]. Long-range electrostatic and dispersion interactions were implemented by the Particle-Mesh-Ewald (PME [25]) summation method with a Fourier spacing of 0.12 nm. For reasons of computational efficiencies, geometric combination rules were used for the long-range dispersion interactions in the Lennard–Jones PME algorithm [27].

The cutoff lengths for the short-range interactions were set to 1.1 nm for AMBER19SB and OPLS-AA/M, and 1.2 nm for CHARMM36m with rvdw-switch/rcoulomb-switch 1 nm. The Potential-shift-Verlet was used to modify the vdW potential for the AMBER19SB and OPLS-AA/M, and a force-switch was used in the case of the CHARMM36m force field. For a comparison of the shifting and switching functions, please see ref. [29]. Unrestrained production dynamics were integrated with a 1 fs time step and a 100–200 ns production time was used for sampling the systems in the NpT ensemble.

### Analysis of Data

Analysis of simulations was done with the GROMACS software suite [62]. Hydrogen bonds were determined using a geometric criterion where a donor-acceptor pair is considered hydrogen bonded if the distance is less than 0.35 nm and the hydrogen-donor-acceptor angle is less than 30°. Isotropic B-factors and angles were calculated using Python (NumPy, and Pandas) [63, 64]. Molecular images were produced using the PyMOL software [65]. Matplotlib was used for generating all plots [66]. The scripts are available from the github repository [56].

## Results and Discussion

Using different atomistic force fields, combined with their recommended water models, we constructed 12 atomic models of peptide crystals. In what follows we analyse the properties of the peptides in relatively long MD simulations.

### Peptide Dynamics in the Crystal Lattice

To quantify the dynamic properties of the peptides, we computed the three-dimensional mean square positional fluctuations (MSF) averaged over the last 50 ns of the simulations (Fig. S1). From the MSF, isotropic B-factors (*B*) were computed using [67]:

1 $$\begin{aligned} B=\frac{8\pi ^2}{3} \left<\textrm{MSF}\right>. \end{aligned}$$

While the MSF of the peptide converges rapidly in the simulation at 100 K, they do not converge within 50 ns at room temperature, which is why all simulations are at least 100 ns. Figure S1 shows the root mean square positional fluctuations for all peptides. In most cases the fluctuations are <0.025 nm at 100 K and <0.1 nm at room temperature, suggesting the peptides move only a little during the simulations. The mean square fluctuations scale roughly with the temperature, as expected. Fig S3 shows the correlation between the experimental and calculated B-factors for the twelve peptides. There is no correlation whatsoever between experimental and calculated isotropic B-factors. Indeed the experimental values are, with some exceptions, systematically higher than the calculated ones (Fig. S2). This suggests that other factors such as crystal mosaicity may lead to the relatively large experimental B-factors [68]. In a simulation study of a protein crystal with different force fields and water models only the Amber99SB force field [69], showed fluctuations comparable to the experimental B-factors[70]. Other than that, there is little evidence to support that simulated B-factors are accurate enough to warrant quantitative evaluation. This, in combination with the lack of correlation, makes further analysis of peptide dynamics not worthwhile.

### Crystal Stability

**Fig. 2:**
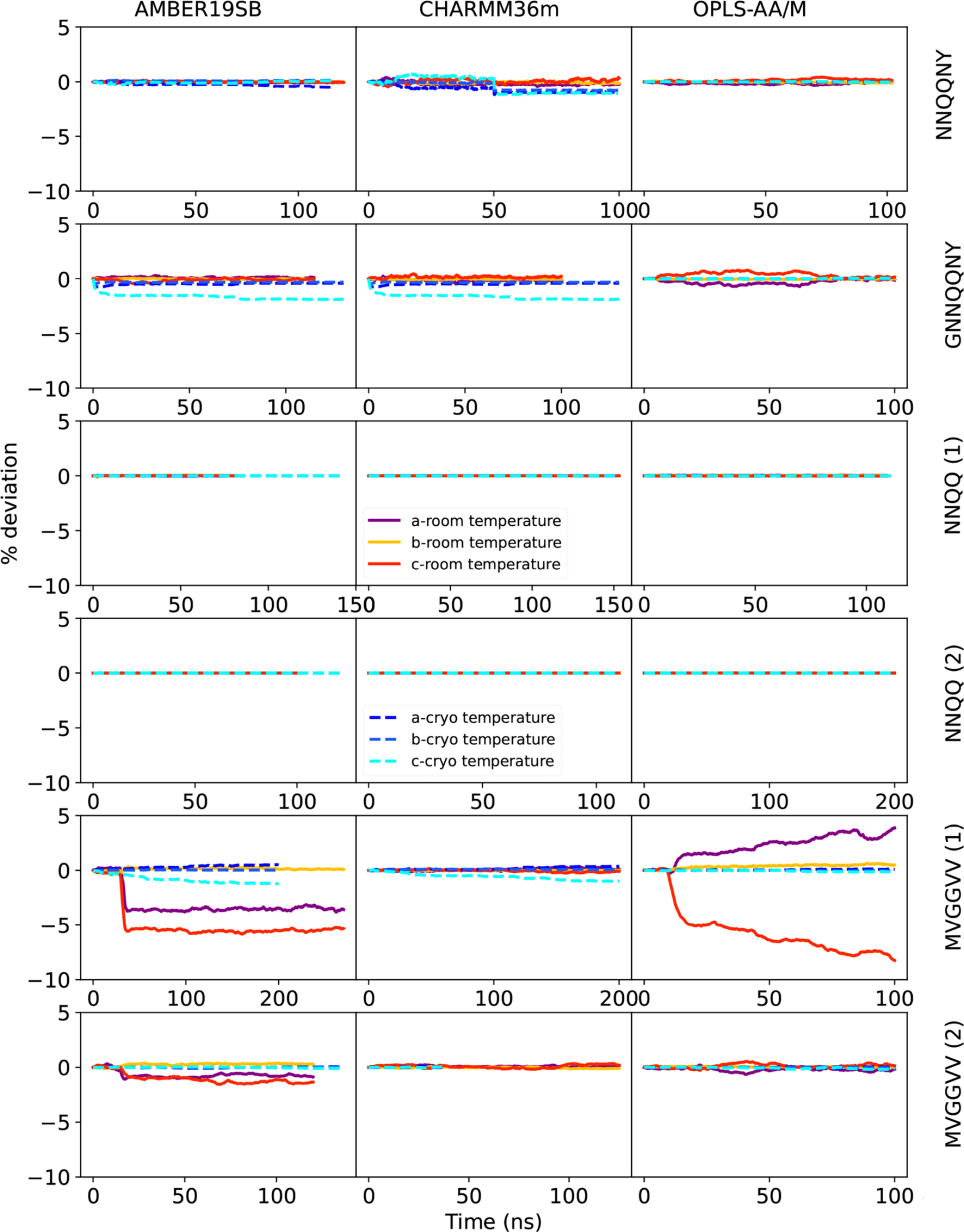
Deviation of lattice size of the supercell in %, over the NpT and production runs for the NNQQNY, GNNQQNY, NNQQ (1), NNQQ (2), MVGGVV (1), and MVGGVV (2) peptides and all three force fields

**Fig. 3:**
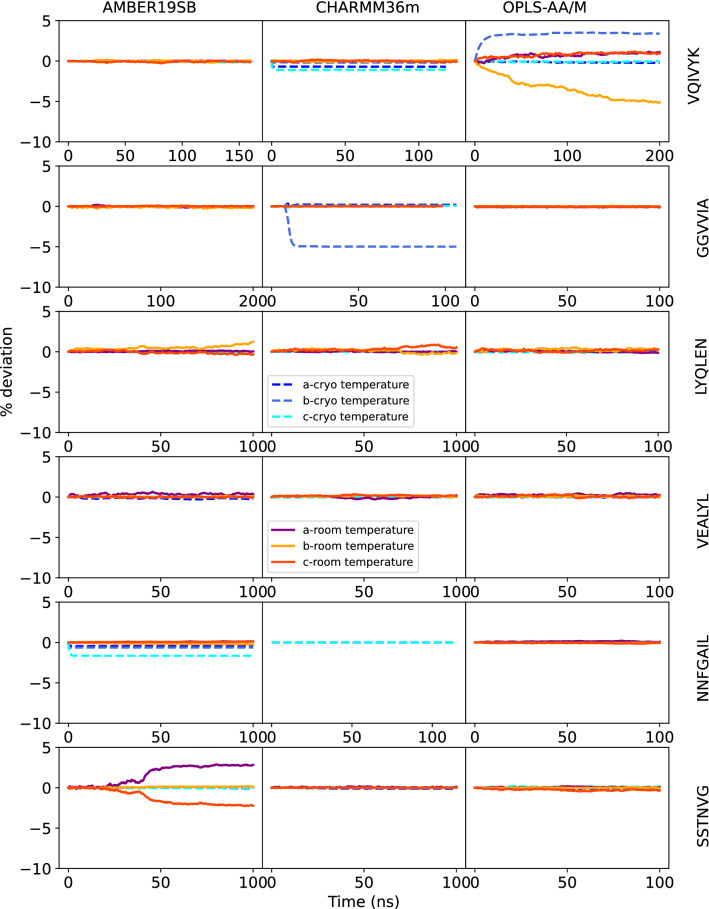
Deviation of lattice size of the supercell in %, over the NpT and production runs for the VQIVYK, GGVVIA, LYQLEN, VEALYL, NNFGAIL, and SSTNVG peptides for all force fields

**Fig. 4:**
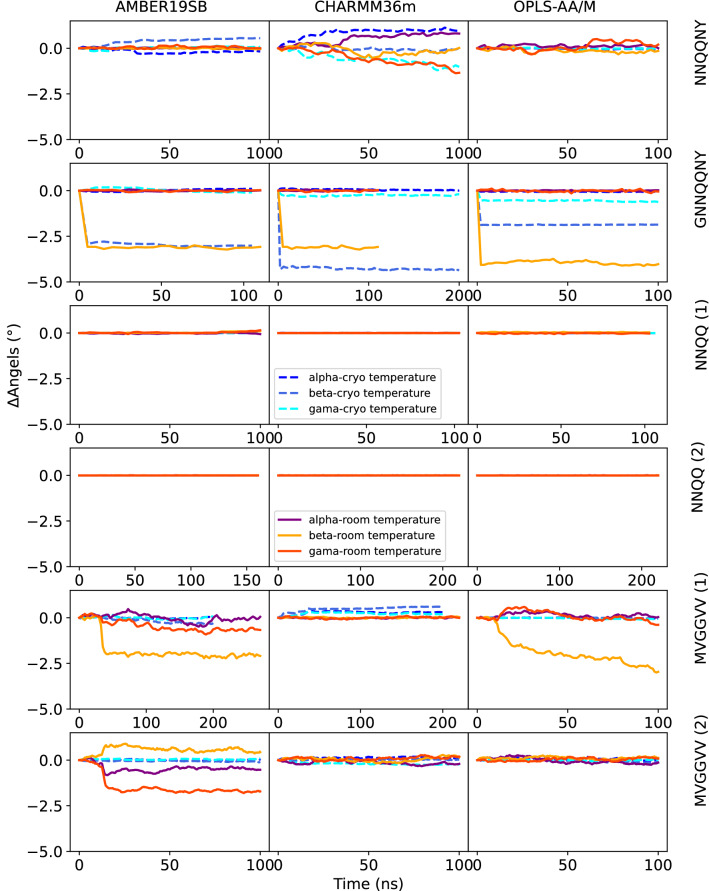
Deviation of angles of the supercell from experimental crystal structure over the NpT and production runs for the NNQQNY, GNNQQNY, NNQQ (1), NNQQ (2), MVGGVV (1), and MVGGVV (2) peptides and all three force fields

**Fig. 5:**
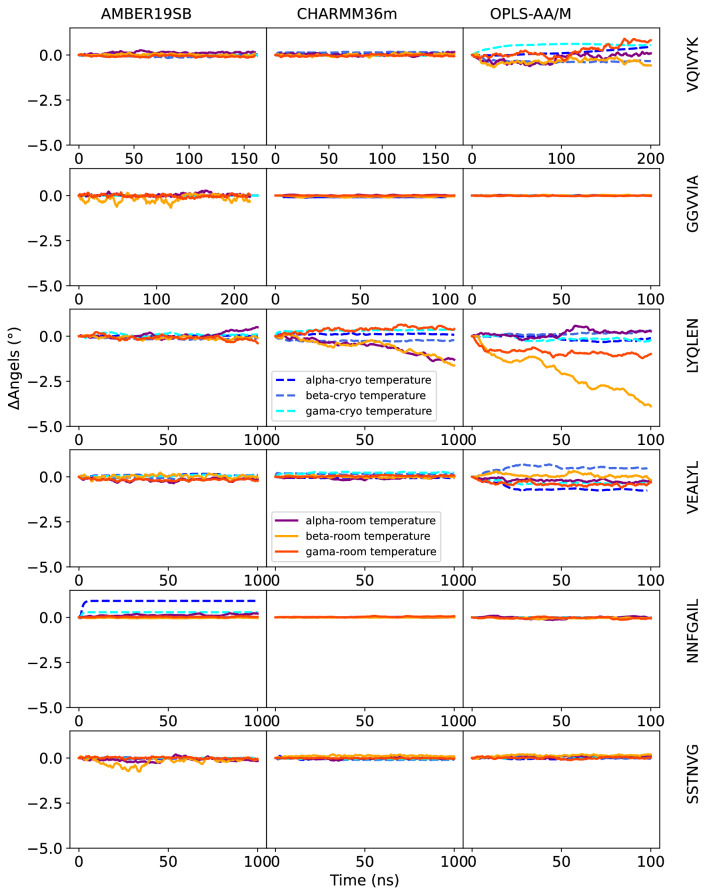
Deviation of angles of the supercell from experimental crystal structure over the NpT and production runs for the VQIVYK, and GGVVIA, LYQLEN, VEALYL, NNFGAIL, and SSTNVG peptides for all force fields

The kinetic stability of the peptide crystals was analyzed by plotting the deviations from the original lattice cell parameters as a function of time. Cell edges a, b, c are plotted in Figs. 2 and 3, angles *α*, *β*, *γ* in Figs. 4 and 5 The mean signed deviation from the original cell parameters is printed in Table S1 for all peptides and force fields. The kinetic stability of the crystals in the simulations depends on peptide sequence, force field, temperature as well as the initial crystal arrangement. The latter can be concluded from the fact that both crystal forms of the NNQQ peptide are very stable, but for the MVGGVV peptide there are differences. The MVGGVV (1) crystal is destabilized in AMBER19SB and OPLS-AA/M (Fig. 2,  4) and, in addition, the MVGGVV (2) crystal is unstable in AMBER19SB. The MVGGVV (1) crystal also has higher MSF than MVGGVV (2) for these two force fields (Fig. S1). It has been suggested that the use of point charges for sulfur atoms, such as in Methionine, does not provide a sufficiently accurate model of the electrostatic properties of the atom [71]. Indeed, Methionine can function both as a hydrophobic residue or as a weakly polar residue, facilitating interactions with a wide variety of other groups [72]. Whether this is part of the explanation of the relatively poor stability of the MVGGVV crystal is difficult to determine based on our results. The GNNQQNY peptide undergoes a rapid change of the *β* angle in all force fields (Fig. 4), not unlike what was seen in density functional theory calculations when water was removed from the crystal [13].

On the other end of the spectrum, the highly polar NNQQ (1) and NNQQ (2) peptides show only small deviations and the unit cell dimensions are maintained during the simulations for all force fields. The peptides containing a charged amino acid (VQIVYK and VEALYL) seem to be stable in all force fields, except for VQIVYK in OPLS-AA/M (Figs.  2-5). The unit cell angles for LYQLEN are unstable for CHARMM36m and OPLS-AA/M (Fig. 5). The supercell of the neutral peptide SSTNVG seems to be unstable in AMBER19SB at room temperature, whereas the GGVVIA peptide crystal is unstable at cryo temperature in the CHARMM36m force field.

### Energetics

Herman Berendsen, whom this article and this journal issue are a tribute to, once remarked that biophyicists are keen to analyse simulated protein structures, while the molecular energy in their simulations is typically ignored. However, energy is often informative, even if it is not a Gibbs energy. Figures S5 and S6 show the time evolution of the total energy (normalized per peptide, including zero or more water molecules) for the cryo and room temperature simulations, respectively. Since classical force fields do not produce directly comparable energies in the same manner as quantum chemistry methods, we subtracted the energy at the start of the simulation is to zero. In this manner, the change in energy during the simulation can be monitored. The changes in unit cell dimensions for certain peptide crystal/force field combinations that were noted above, typically coincide with a drop in energy. NNQQ (1) and (2) show very stable total energy over time in all force fields at room and cryo temperatures, which could be due to their enthalpically favored structures (Figs. S5 and S6). LYQLEN at room temperature with AMBER19SB and CHARMM36m, NNQQNY with AMBER19SB at 293 K, VQIVYK with AMBER19SB and CHARMM36m at room 291 K, VEALYL at 100 K with AMBER19SB parameter, GNNQQNY at 291 K with AMBER19SB, VQIVYK at 100 K with AMBER19SB and CHARMM36m, GNNQQNY at cryo temperate using OPLS-AA/M, and NNFGAIL at 100 K with all force field parameters show very converged total energies. While in the case of MVGGVV (1) at cryo temperature using CHARMM36m, VEALYL with OPLS-AA/M at 100 K, VEALYL with CHARMM36m at 310 K, MVGGVV (2) at 298 K with AMBER19SB, VQIVYK at 291 K with OPLS-AA/M, SSTNVG at 100 K with AMBER19SB, and LYQLEN at room temperature with OPLS-AA/M, show that the total energies do not convergence entirely during the simulations. Taken together, for all force fields there is at least one peptide where the total energy is not converged. To reach equilibrium in crystals, longer simulation time is needed than in solution simulations, since the energy barriers for conformational change are exacerbated by the periodic boundary conditions. Ultimately, the Gibbs energy of the peptide crystals that is modeled by the force fields determines the changes in unit cell dimension. Although the changes in energy are a few kJ/mol only in most cases, there is an entropic component to the Gibbs energy that is difficult to estimate from our simulations. We therefore proceed with what at least some simulation biophysicists prefer to do.

**Table 3:** Average change in main-chain hydrogen bonding per peptide during the last 50 ns simulations with the crystal structure as a reference

Sequence	T (K)	AMBER19SB	CHARMM36m	OPLS-AA/M
NNQQNY	293	0.3	−0.1	−0.1
NNQQNY	100	0.0	0.1	0.1
GNNQQNY	293	0.0	0.0	−0.1
GNNQQNY	100	−0.7	0.0	−0.0
NNQQ (1)	298	0.2	−0.0	0.1
NNQQ (1)	100	0.2	0.1	0.2
NNQQ (2)	298	0.1	0.2	0.2
NNQQ (2)	100	0.2	0.2	0.3
GGVVIA	291	0.1	−0.0	0.2
GGVVIA	100	0.5	−0.1	−0.1
MVGGVV (1)	298	0.2	−0.1	−0.5
MVGGVV (1)	100	0.0	−0.0	−0.2
MVGGVV (2)	291	0.1	−0.7	−0.3
MVGGVV (2)	100	0.8	−0.4	0.1
VQIVYK	291	−0.0	−0.0	−0.1
VQIVYK	100	−0.0	0.1	0.3
LYQLEN	310	−0.2	0.4	0.1
LYQLEN	100	0.5	0.2	−0.2
VEALYL	310	0.0	0.6	0.5
VEALYL	100	0.2	0.8	0.1
NNFGAIL	293	0.4	0.2	0.1
NNFGAIL	100	0.8	−0.2	−0.1
SSTNVG	293	0.0	0.0	0.4
SSTNVG	100	0.2	−0.0	−0.0

### Detailed Structural Analysis

Intermolecular hydrogen bonding between the amide and carbonyl groups of main chains has a key role in stabilizing protein tertiary structure, including amyloid fibrils [73]. Table S2 shows that the number of hydrogen bonds is comparable between the three force fields, in all cases slightly overestimating the number of hydrogen bonds involving peptides compared to the crystal structures. This is particularly apparent in the three “dry” crystals, NNQQ (1), NNQQ (2) and MVGGVV (1), that have no water at all, but where nevertheless the number of hydrogen bonds increases. A similar finding was reported by Cerutti and co-workers, who explored the excessive formation of hydrogen bonds in simulations of protein crystals using several different proteins force fields and water models [70]. Water-water hydrogen bonds are reduced somewhat in all cases which seems to be due to an increased number H-bonds between water and peptides. Table 3 lists the change in main-chain (backbone + amide hydrogen) hydrogen bonds during the simulations, i.e. those hydrogen bonds that are involved in the tertiary structure. In most cases, the changes are small, in part because the numbers are averaged over all peptides in the supercell.

To investigate whether the peptides stay in their crystal conformation during the simulations, we have calculated the backbone *ϕ*/*ψ* angles, i.e. the Ramachandran plots [47] (Figs. S5 and S6). Since the peptides initially are in predominantly *β* strand conformation, most angles should be in the area close to −120°/−120°. The MVGGVV peptides lose their structure in the simulations (Fig. S5) to varying degrees in all force fields and this change is correlated to the change in energy (Figs. S3 and S4) and a change in simulation cell size (Fig. 2).

For the case of LYQLEN, both the Ramachandran plot (Fig. S6) and the deviation of unit cell angles when using the OPLS-AA/M force field (Fig. 5) show the peptide crystal is not stable, to a larger extent than when using AMBER19SB or CHARMM36m. The VQIVYK crystal is less stable when using OPLS-AA/M than with other force fields. These results show the difficulty of using one set of force field parameters for the simulation of peptide crystals suggesting continued development of the force fields is needed. We should point out other works that showed that it is difficult to reproduce *ϕ*/*ψ* angles, i.e., a Ramachandran plot in simulations in aqueous solution as well [74, 75].

**Table 4:** Mean signed deviation from crystal parameters as a function of temperature for the three force fields studied

Property	T(K)	AMBER19SB	CHARMM36m	OPLS-AA/M
Box edge (%)	100	−0.2	−0.3	0.1
	RT	−0.2	0.0	−0.1
Angle (°)	100	0.0	0.1	0.1
	RT	0.2	0.1	−0.2
Volume (%)	100	−0.2	−1.0	0.3
	RT	−0.7	0.1	−0.4
HB P-P (%)	100	105.5	108.7	104.7
	RT	99.8	104.3	100.8
HB P-W (%)	100	105.7	113.0	100.6
	RT	98.5	97.6	101.4
HB W-W (%)	100	59.0	55.3	62.9
	RT	51.1	58.0	47.4

. Box edge, averaged over a, b, c. Angle averaged over *α*, *β*, *γ*. Peptide-peptide hydrogen bonds (HB P-P), Peptide-water hydrogen bonds (HB P-W), and water-water hydrogen bonds (HB W-W) as a percentage of the number in the crystal structure. RT indicates the crystallization temperature, see Table 1

## Conclusions

The pathological hallmark of amyloid diseases is the formation of amyloid fibrils. Much of the literature on amyloid fibril structure, formation and dynamics is related to thermodynamics, in a manner similar to the protein folding problem [7]. In short, amyloid formation can be described as a solubility problem where proteins aggregate if the concentration exceeds a critical concentration locally. It is interesting to note that chaperone proteins have been shown to bind to fibrils and dissolve them [76] in a manner similar to what happens in in vivo protein folding, where chaperones bind to partially folded proteins in order to prevent misfolding. Obviously, the fibrillization process in vivo is more complicated than what is tractible in vitro, let alone in silico [22].

Even though it should be acknowledged that theoretical models cannot describe the complex amyloid biology, it is reasonable to expect that models based on physics, such as force fields, should be able to simulate peptide crystals without altering the peptide structure. Table 4 summarizes the deviations from the observables analyzed in this work. Most deviations from unit cell parameters are close to zero, and the number of hydrogen bonds involving peptides is close to 100% of what is found in the crystal. This is corroborated by the Ramachandran plots (Figs. S5, S6) that show that most peptides indeed remain stable in the simulations. However, there are one or more exceptions for all force fields, consistent with the work of Janowski et al. [43], suggesting that further work on force fields is needed before reliable predictions on amyloid stability and formation can be made.

## Supplementary Information

Supplementary tables with deviation from unit cell dimensions as well as hydrogen bond information are available. Supplementary figures of root mean square fluctuations as well as B-factors and energies are available.

## Supplementary Information

Below is the link to the electronic supplementary material.

Supplementary file1 (PDF 18,925 KB)
