# Supplementary material for: Simulations of Amyloid-Forming Peptides in the Crystal State
Source: Protein J. 2023 May 5;42(3):192–204. doi: 10.1007/s10930-023-10119-3 (PMC10264543; doi:10.1007/s10930-023-10119-3)
Supplement: Supplementary file 1 — Supplementary file1 (PDF 18,925 KB) [file 10930_2023_10119_MOESM1_ESM.pdf]

Simulations of Amyloid-forming Peptides in the Crystal State - Supplementary Information

A. Najla Hosseini[1] and David Van der Spoel[1]

[1]Department of Cell and Molecular Biology, Uppsala University, Box 596, SE-75124Uppsala, Sweden.

Contributing authors: David.VanderSpoel@icm.uu.se;

Table S1 Mean signed deviation from lattice parameters a,b,c (%) and angles α,β,γ (°) averaged over the last 50 ns of the simulation at RT and 100 K.

Peptide		AMBER19SB						CHARMM36m						OPLS-AA/M					
	T (K)	a	b	c	α	β	γ	a	b	c	α	β	γ	a	b	c	α	β	γ
NNQQNY	293	-0.1	0.0	0.0	0.0	-0.0	0.1	-0.2	-0.1	0.0	0.5	-0.1	-0.6	-0.2	-0.0	0.1	0.1	-0.1	0.1
NNQQNY	100	-0.1	0.0	0.0	-0.2	0.4	0.0	-0.7	-0.4	-0.4	0.8	-0.0	-0.6	-0.0	-0.0	0.0	-0.0	-0.0	-0.0
GNNQQNY	293	0.1	-0.0	-0.1	-0.0	-3.0	0.0	-0.1	-0.0	0.2	-0.0	-4.5	0.1	-0.3	-0.0	0.4	0.0	-3.9	-0.0
GNNQQNY	100	-0.5	-0.3	-1.7	0.0	-2.8	0.0	-1.0	-0.4	-1.7	0.1	-4.2	-0.3	0.0	0.0	0.0	0.0	-1.8	0.0
NNQQ (1)	298	-0.0	0.0	0.0	-0.0	0.0	0.0	0.0	-0.0	0.0	0.0	0.0	-0.0	0.0	0.0	-0.0	0.0	0.0	-0.0
NNQQ (1)	100	-0.0	-0.0	0.0	0.0	-0.0	-0.0	-0.0	-0.0	0.0	-0.0	-0.0	0.0	0.0	0.0	0.0	-0.0	-0.0	0.0
NNQQ (2)	298	-0.0	-0.0	0.0	-0.0	0.0	-0.0	-0.0	0.0	0.0	-0.0	0.0	-0.0	0.0	0.0	0.0	-0.0	-0.0	-0.0
NNQQ (2)	100	0.0	0.0	0.0	-0.0	-0.0	-0.0	0.0	0.0	0.0	-0.0	0.0	-0.0	-0.0	-0.0	-0.0	-0.0	-0.0	-0.0
GGVVIA	291	-0.0	-0.1	0.0	0.0	-0.2	-0.0	0.0	0.0	0.0	0.0	-0.1	0.0	-0.1	0.0	-0.0	0.0	0.0	-0.0
GGVVIA	100	-0.0	-0.0	-0.0	0.0	-0.0	-0.0	0.2	-4.5	0.1	-0.1	-0.0	-0.0	-0.0	-0.0	-0.0	0.0	0.0	0.0
MVGGVV (1)	298	-3.1	0.1	-4.9	-0.0	-1.8	-0.5	0.1	0.0	-0.0	-0.0	0.0	0.0	2.2	0.4	-5.5	0.1	-1.8	0.1
MVGGVV (1)	100	0.3	0.0	-0.8	0.0	-0.2	-0.0	0.2	0.1	-0.6	0.3	0.5	0.2	0.0	-0.0	-0.1	-0.0	-0.0	-0.0
MVGGVV (2)	291	-0.7	0.3	-1.0	-0.5	0.6	-1.4	0.1	-0.0	0.1	-0.1	0.0	0.1	-0.2	0.0	0.1	-0.0	0.1	0.0
MVGGVV (2)	100	-0.0	0.0	-0.0	0.0	-0.1	0.0	0.0	-0.0	0.0	0.1	0.0	-0.2	-0.0	0.0	-0.1	0.1	-0.0	-0.0
VQIVYK	291	-0.0	0.0	-0.1	0.1	0.0	-0.1	-0.0	-0.0	0.0	0.0	0.0	-0.0	0.7	-3.4	0.8	-0.2	-0.3	0.2
VQIVYK	100	0.0	-0.0	-0.0	-0.0	-0.1	-0.0	-0.7	-0.2	-1.1	0.0	0.1	-0.0	-0.1	3.3	-0.1	0.1	-0.4	0.5
LYQLEN	310	0.1	0.5	-0.1	0.1	-0.1	-0.1	0.0	0.1	0.4	0.0	-0.1	0.1	0.0	0.3	0.1	0.1	-2.0	-0.9
LYQLEN	100	0.0	0.0	0.0	0.0	-0.1	0.1	-0.0	0.0	-0.0	0.1	-0.3	0.3	0.1	-0.0	-0.1	-0.1	0.1	-0.1
VEALYL	310	0.4	0.1	0.0	-0.2	0.0	-0.2	-0.0	0.1	0.2	-0.1	-0.0	0.0	0.3	0.1	0.1	-0.2	0.1	-0.4
VEALYL	100	-0.2	-0.0	0.0	0.1	-0.1	0.1	-0.0	0.0	-0.0	-0.1	0.2	0.2	-0.0	0.0	-0.0	-0.6	0.5	-0.3
NNFGAIL	293	0.1	-0.1	0.1	0.1	-0.0	0.0	-0.0	-0.0	-0.0	0.0	-0.0	0.0	0.1	-0.0	-0.1	-0.0	-0.0	-0.0
NNFGAIL	100	-0.5	-0.6	-1.6	0.9	0.0	0.3	-0.0	0.0	0.0	-0.0	-0.0	-0.0	0.0	-0.0	-0.0	-0.0	-0.0	-0.0
SSTNVG	293	1.7	0.1	-1.2	-0.1	-0.2	0.0	0.1	0.0	0.0	-0.0	0.1	-0.0	0.1	0.0	-0.3	0.1	0.1	0.0
SSTNVG	100	-0.0	0.0	-0.0	-0.0	0.0	-0.0	-0.1	0.0	0.1	-0.1	-0.1	-0.1	-0.1	-0.0	0.1	-0.0	0.0	0.0

Table S2 Number of hydrogen bonds averaged over the last 50 ns of the production simulation compared to the crystal structure. P: peptide, W: water. A minus sign indicates no water is present.

Sequence		X-ray			AMBER19SB			CHARMM36m			OPLS-AA/M		
	T (K)	P-P	P-W	W-W	P-P	P-W	W-W	P-P	P-W	W-W	P-P	P-W	W-W
NNQQNY	293	14	5	8	12.3	9.9	6.0	11.4	8.9	5.3	12.3	9.2	5.0
NNQQNY	100	14	5	8	12.1	10.0	6.8	12.2	9.7	5.9	12.7	9.3	6.1
GNNQQNY	293	16	8	6	14.7	8.4	7.0	14.3	8.8	6.8	15.1	8.0	7.6
GNNQQNY	100	16	8	6	15.4	9.2	7.9	15.1	9.5	7.9	15.6	7.8	7.6
NNQQ (1)	298	10	-	-	11.0	-	-	12.0	-	-	11.8	-	-
NNQQ (1)	100	10	-	-	11.5	-	-	13.4	-	-	12.2	-	-
NNQQ (2)	298	14	-	-	11.5	-	-	11.9	-	-	11.9	-	-
NNQQ (2)	100	14	-	-	12.8	-	-	12.9	-	-	12.6	-	-
GGVVIA	291	7	4	2	6.2	3.7	2.1	6.2	3.7	2.2	6.0	3.7	2.1
GGVVIA	100	7	4	2	6.6	5.3	2.2	6.8	5.0	2.6	6.0	4.0	2.5
MVGGVV (1)	298	6	-	-	6.7	-	-	7.0	-	-	6.7	-	-
MVGGVV (1)	100	6	-	-	6.8	-	-	7.2	-	-	6.9	-	-
MVGGVV (2)	291	7	2	1	6.6	2.5	0.4	6.6	2.6	0.6	7.04	2.3	0.5
MVGGVV (2)	100	7	2	1	7.2	2.4	0.3	6.8	2.8	0.5	7.1	2.5	0.6
VQIVYK	291	10	3	3	10.4	5.2	2.5	10.4	5.2	2.5	7.2	6.7	1.3
VQIVYK	100	10	3	3	10.1	6.1	2.6	11.1	5.4	2.7	8.5	5.0	2.6
LYQLEN	310	7	5	2	8.0	6.1	0.9	8.8	5.9	1.1	8.4	6.2	0.8
LYQLEN	100	7	5	2	9.6	4.5	2.3	8.5	6.8	1.0	8.5	5.9	1.9
VEALYL	310	6	2	0	6.4	1.6	0.2	7.8	1.4	0.3	7.1	1.6	0.1
VEALYL	100	6	2	0	6.9	1.4	0.5	8.2	1.4	0.3	7.1	2.0	0.2
NNFGAIL	293	11	3	0	9.5	3.0	0.0	9.6	3.0	0.0	9.8	2.3	0.0
NNFGAIL	100	11	3	0	10.6	3.0	0.0	10.7	2.5	0.0	10.8	2.9	0.0
SSTNVG	293	9	3	2	9.7	4.4	1.8	10.4	4.8	2.3	10.1	5.3	1.5
SSTNVG	100	9	3	2	9.2	5.9	1.6	9.2	7.8	1.0	10.4	5.2	2.1

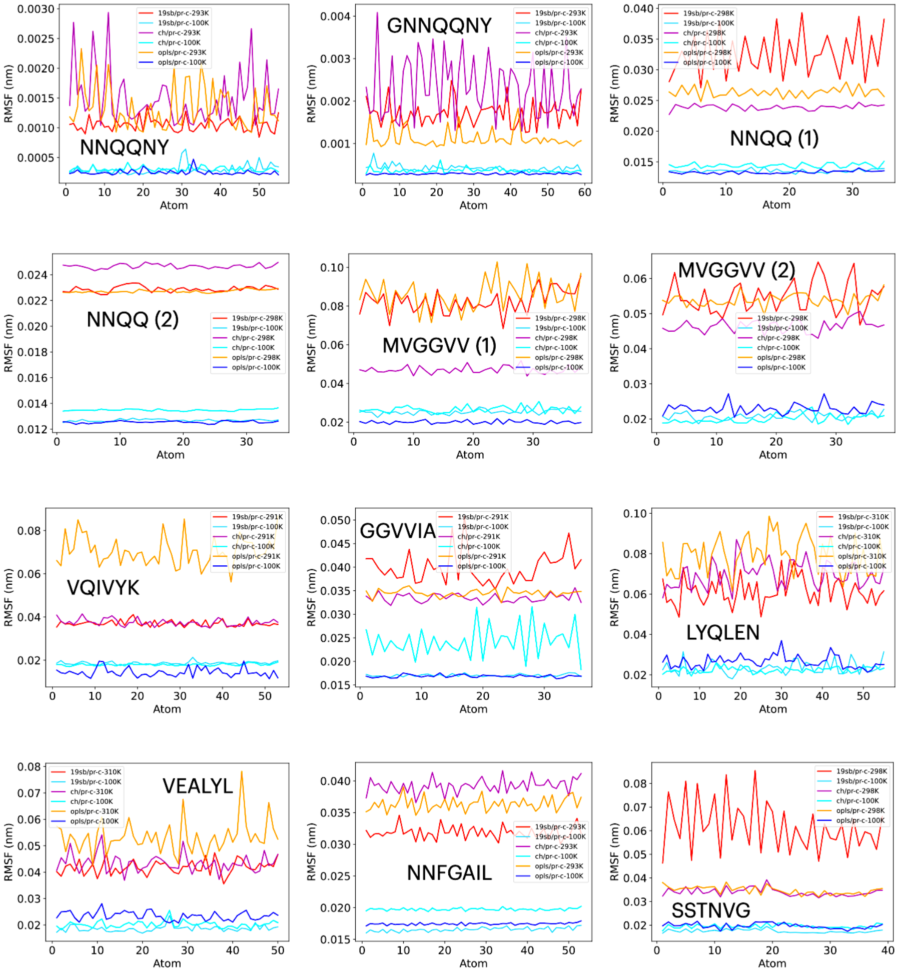

Fig. S1 Root mean square fluctuations from atomic positions averaged over the last 50 ns of the simulations.

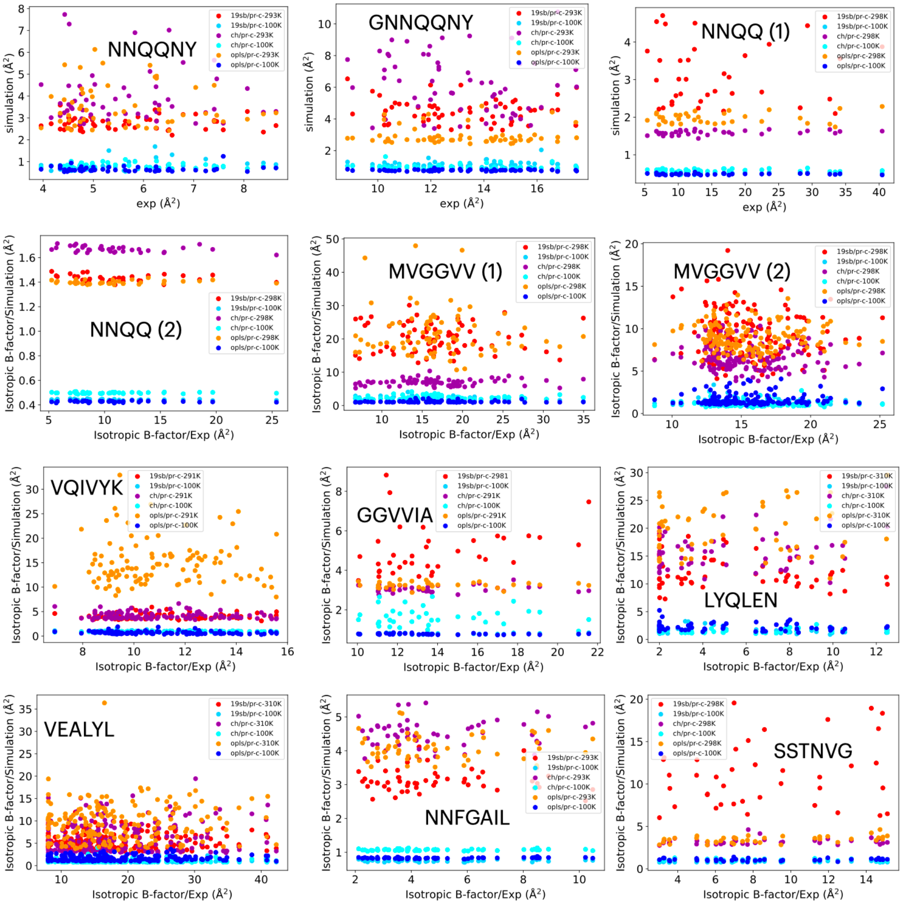

Fig. S2 Correlation between experimental (X-axis) and calculated (Y-axis) B-factors.

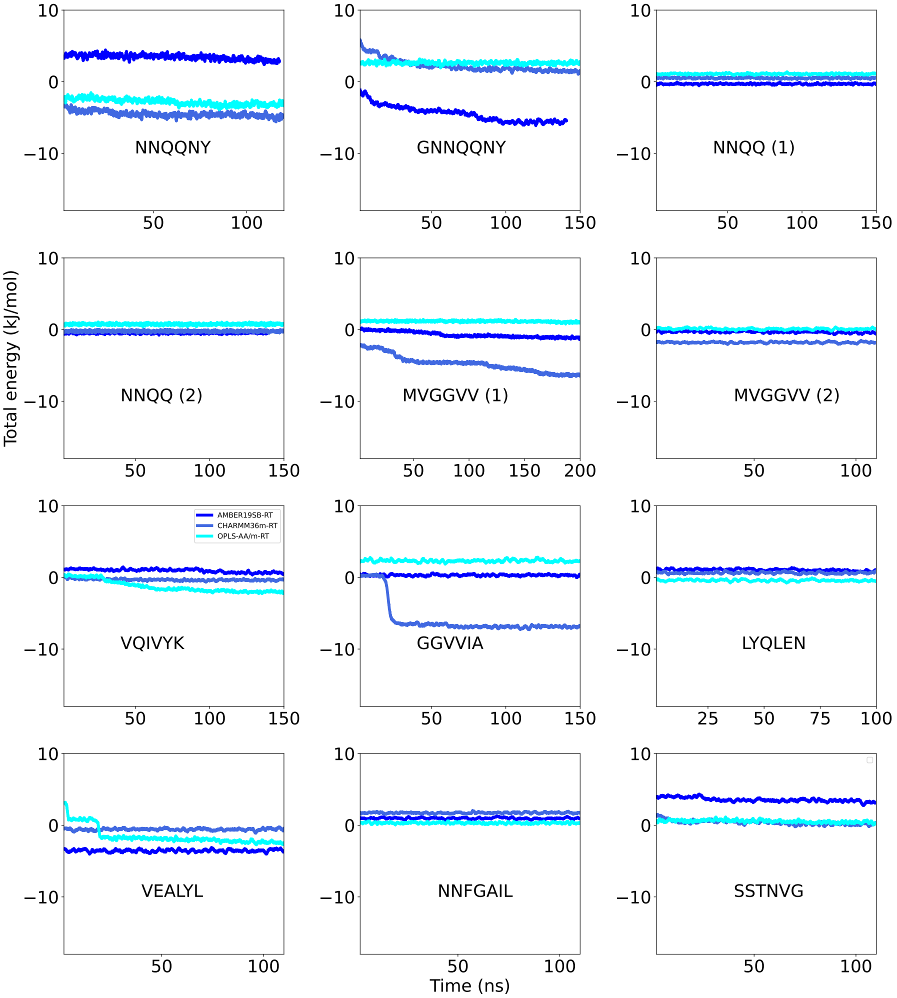

Fig. S3 Total energy per peptide relative to the starting energy for the simulations at 100K.

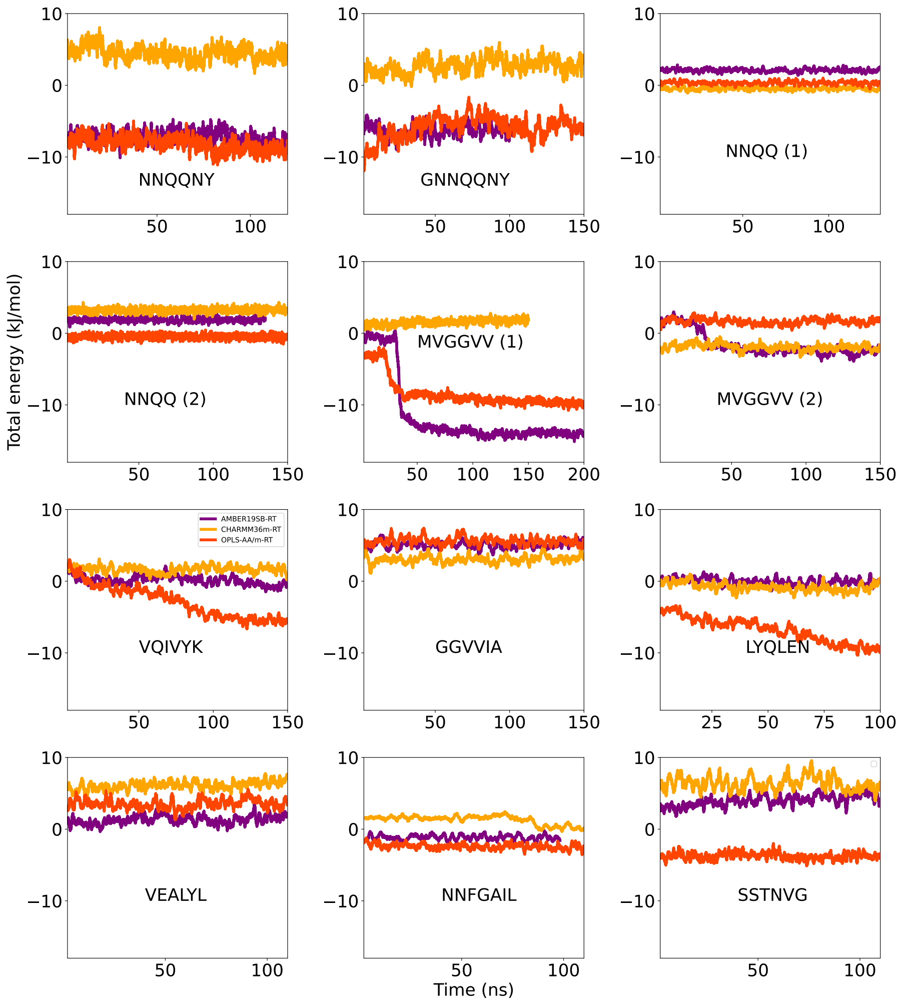

Fig. S4 Total energy per peptide relative to the starting energy for the simulations at room temperature.

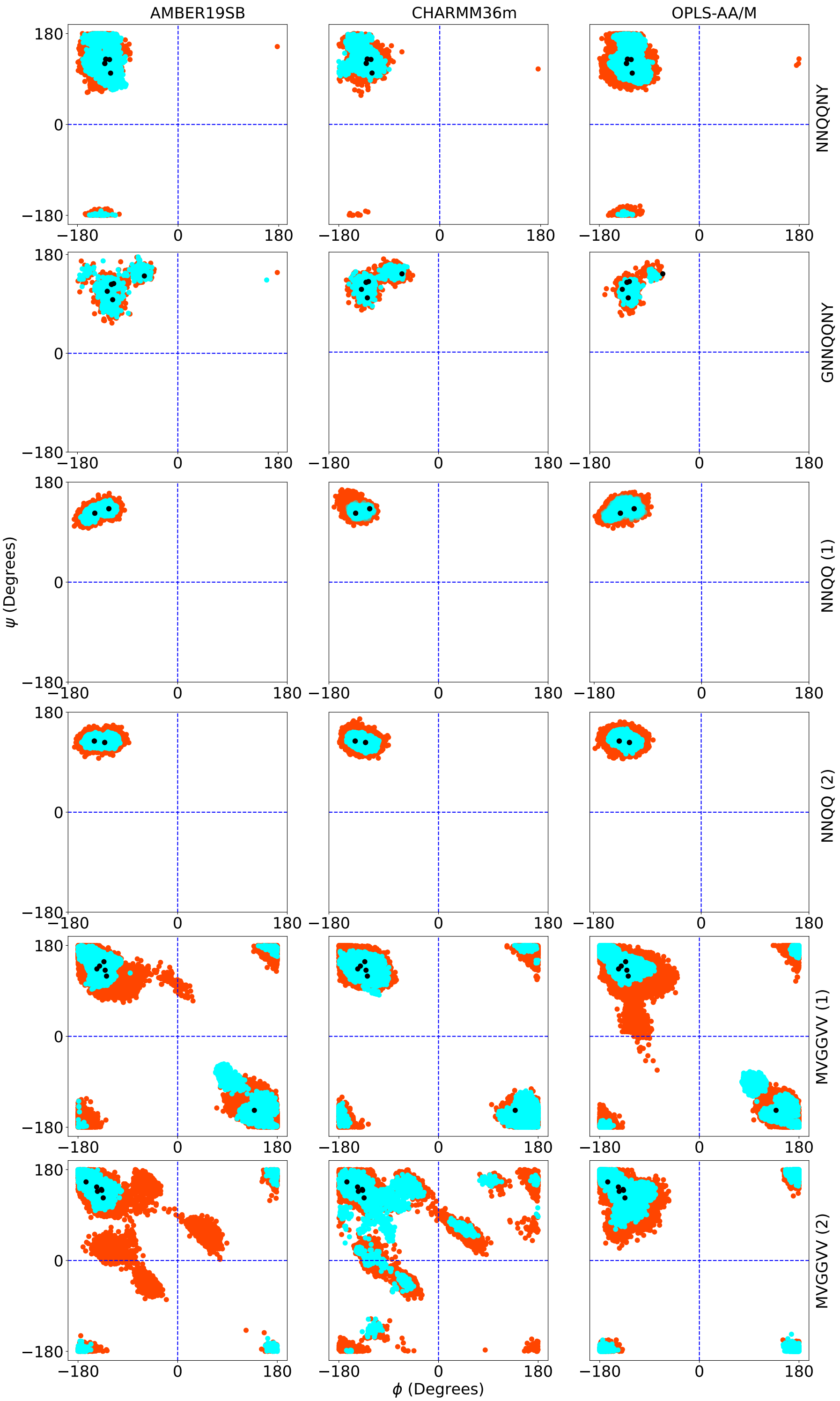

Fig. S5 Ramachandran plots for the NNQQNY, GNNQQNY, NNQQ (1), NNQQ (2), MVGGVV (1), and MVGGVV (2) peptides and all three force fields.

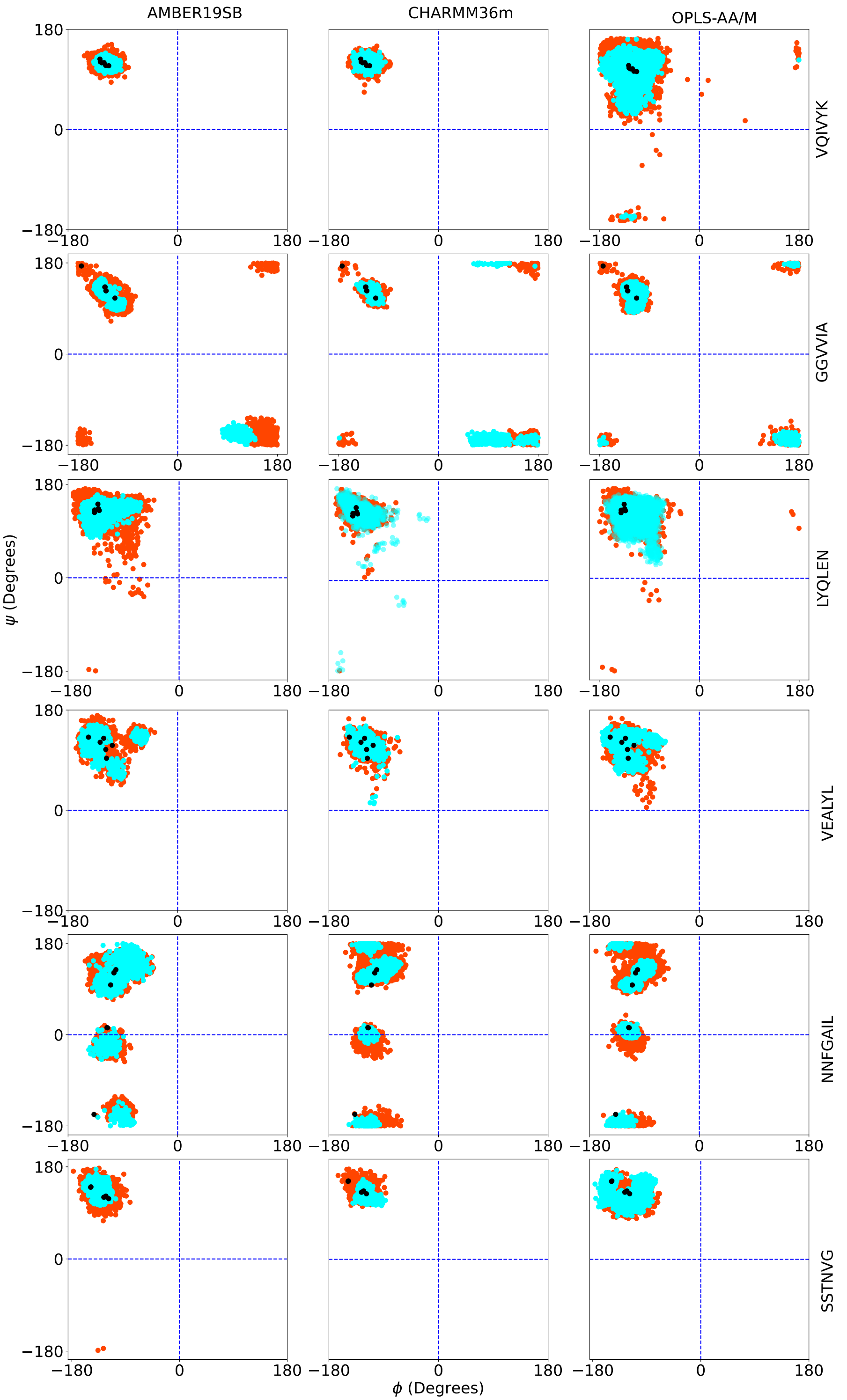

Fig. S6 Ramachandran plots for the VQIVYK, GGVVIA, LYQLEN, VEALYL, NNFGAIL, and SSTNVG peptides and all three force fields.
